# The Protective Effects of Ivabradine in Preventing Progression from Viral Myocarditis to Dilated Cardiomyopathy

**DOI:** 10.3389/fphar.2016.00408

**Published:** 2016-11-01

**Authors:** Li Yue-Chun, Chen Guang-Yi, Ge Li-Sha, Xing Chao, Tian Xinqiao, Lin Cong, Dai Xiao-Ya, Yang Xiangjun

**Affiliations:** ^1^Department of Cardiology, First Affiliated Hospital of Soochow UniversitySuzhou, China; ^2^Department of Cardiology, Second Affiliated Hospital of Wenzhou Medical UniversityWenzhou, China; ^3^Department of Pediatrics, Second Affiliated Hospital of Wenzhou Medical UniversityWenzhou, China; ^4^Department of Clinical Laboratory, Second Affiliated Hospital of Wenzhou Medical UniversityWenzhou, China; ^5^Department of Ultrasonography, Henan Provincial People’s Hospital (People’s Hospital of Zhengzhou University), ZhengzhouChina

**Keywords:** ivabradine, chronic viral myocarditis, cytokines, fibrosis, p38 MAPK

## Abstract

To study the beneficial effects of ivabradine in dilated cardiomyopathy (DCM) mice, which evolved from coxsackievirus B3-induced chronic viral myocarditis. Four-to-five-week-old male balb/c mice were inoculated intraperitoneally with coxsackievirus B3 (Strain Nancy) on days 1, 14, and 28. The day of the first virus inoculation was defined as day 1. Thirty-five days later, the surviving chronic viral myocarditis mice were divided randomly into two groups, a treatment group and an untreated group. Ivabradine was administered by gavage for 30 consecutive days in the treatment group, and the untreated group was administered normal saline. Masson’s trichrome stain was used to evaluate the fibrosis degree in myocardial tissue. The expression levels of tumor necrosis factor-α (TNF-α), interleukin-1β (IL-1β), interleukin-6 (IL-6), collagen I, collagen III and p38-MAPK signaling pathway proteins were detected by Western blot. Electrocardiogram was used to investigate the heart rate and rhythm. The thickness of the ventricular septum and left ventricular posterior wall, left ventricular end diastolic dimension, left ventricular end systolic dimension, left ventricular ejection fractions and fractional shortening were studied by echocardiography. Compared with the untreated chronic viral myocarditis mice, ivabradine significantly increased the survival rate, attenuated the myocardial lesions and fibrosis, improved the impairment of the left ventricular function, diminished the heart dimension, decreased the production of collagen I and collagen III, reduced the expression of the proinflammatory cytokines TNF-α, IL-1β, and IL-6, and lowered the production of phospho-p38 MAPK. The findings indicate the therapeutic effect of ivabradine in preventing the progression from viral myocarditis to DCM in mice with chronic viral myocarditis induced by coxsackievirus B3, is associated with inhibition of the p38 MAPK pathway, downregulated inflammatory responses and decreased collagen expression. Ivabradine appears a promising approach for the treatment of patients with viral myocarditis.

## Introduction

Coxsackievirus B_3_ (CVB3), considered one of the most widespread causative factors of viral myocarditis, induced more than half of the cases of acute myocarditis and one-fourth of the cases of dilated cardiomyopathy (DCM) (Henke et al., 2003). Acute viral myocarditis would evolve to the chronic stage if there is no effective treatment, and the worst outcome is DCM and chronic heart failure (HF), even cardiac sudden death.

The heart rate (HR) has been shown to be directly related to the risk of cardiovascular disease, HF, and mortality (Opasich et al., 2001). An elevated HR might limit ventricular diastolic filling and increase myocardial oxygen demand. In addition, an increased HR is known to be associated with systemic inflammation and cytokines (Sajadieh et al., 2004). Several experimental studies (Sajadieh et al., 2004; Fox et al., 2008; Perret-Guillaume et al., 2009) have demonstrated that inflammation has a close relationship with HR. With an increased HR, inflammation would be aggravated, and the cardiovascular death rate and hospital admissions because of HF are significantly increased (Sajadieh et al., 2004; Fox et al., 2008; Dominguez-Rodriguez et al., 2012; O Hartaigh et al., 2013).

Ivabradine (IVA), a selective I_f_ channel inhibitor affecting the sinus node, inhibits the pacemaker current and reduces the HR without affecting blood pressure, cardiac contractility and the conduction system (Swedberg et al., 2010). IVA has a beneficial effect in patients with HF (Custodis et al., 2008; Walcher et al., 2010; Dominguez-Rodriguez et al., 2012). Several studies have reported that ivabradine might play a protective role in cardiovascular inflammation. Becher et al. (2012) suggested that ivabradine reduced the expression of cytokines in hypertensive HF, reduced cardiac collagen accumulation and eliminated cardiac fibrosis. Recently, we demonstrated that ivabradine decreased the HR and the expression of pro-inflammatory cytokines and provided protection in a murine model of acute viral myocarditis (Yue-Chun et al., 2012; Li et al., 2013). However, the protective role of ivabradine in DCM caused by chronic viral myocarditis remains unclear. Therefore, in the present study, we investigated if ivabradine attenuated inflammation and prevented the progression from viral myocarditis to DCM in mice with chronic viral myocarditis induced by coxsackievirus B3.

## Materials and Methods

### Chronic Viral Myocarditis Model

**FIGURE 1:**
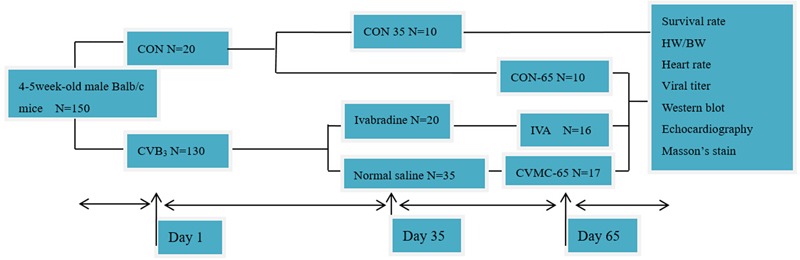
**Experimental design.** CON represents the normal control group. CON-35 and CON-65 represent the normal control groups whose mice were killed on days 35 and 65, respectively. CVB3 represents the chronic viral myocarditis group.

One hundred and fifty 4–5-week-old, male Balb/c mice were purchased from the Shanghai Laboratory Animal Central, China. The mice were first randomly assigned to three groups: normal control group 1 (CON-35, *n* = 10), normal control group 2 (CON-65, *n* = 10), and chronic viral myocarditis group (CVMC, *n* = 130). All mice in the CON-35 and CON-65 groups were killed on days 35 and 65, respectively. The CVMC group mice were inoculated intraperitoneally with 100 TCID_50_ CVB3 (strain Nancy) 0.2, 0.25, and 0.3 ml on days 1, 14, and 28, respectively. We defined the day of the first virus inoculation as day 1. The normal control mice were inoculated intraperitoneally with an equivalent dose of Eagle’s MEM. CVB3 was provided by the American Type Culture Collection (ATCC). The development of chronic viral myocarditis and DCM was assessed, and seven mice from the chronic viral myocarditis group were killed on day 35 (CVMC-35, *n* = 7). Then, the remaining surviving chronic myocarditis mice in the CVMC group were divided into two groups randomly on day 35: an untreated chronic viral myocarditis group (CVMC-65, *n* = 35) and an ivabradine treatment group (IVA, *n* = 20). The treatment group was treated by intragastric administration with ivabradine (5 mg/kg per day) for 30 days consecutively, and the untreated group mice were given a gavage of normal saline of the same volume. IVA was obtained from Servier Co (Courbevoie, France). The doses were selected according to previous experiments (Yue-Chun et al., 2012; Li et al., 2013). The experimental design is shown in **Figure 1**. The study conformed to the China Animal Protection Law. The Wenzhou Medical University Committee on Ethics in the Use and Care of Laboratory Animals approved the conduct of this study.

### Electrocardiogram

The mice were anesthetized intraperitoneally with 4% chloral hydrate (0.02 ml/g) before recording. HR and rhythm were recorded by a Labchat electrocardiogram (ECG) recorder, which was provided by Wenzhou Medical University. ECG was recorded for 1 min continuously.

### Echocardiogram

A Sonos 5500 ultrasound machine (Phillips, USA) was used to perform the transthoracic echocardiography, as described in our previous publications (Yue-Chun et al., 2012; Li-Sha et al., 2015). The mice were anesthetized intraperitoneally with 4% chloral hydrate (0.02 ml/g). The chest was shaved. The parasternal long-axis view was recorded by the M-mode and two-dimensional echocardiographic images. Each of these captured image loops included 5–10 cardiac cycles, and the data were averaged from at least three cycles per loop. The thickness of the ventricular septum and left ventricle posterior wall (LVPW), left ventricular end-systolic diameter (LVESD), and left ventricular end-diastolic diameters (LVEDD) were detected for at least five consecutive cardiac cycles. Then, the left ventricular ejection fraction (LVEF) and fractional shortening (FS) were calculated. End diastolic or end systolic was defined as the maximum or minimal LV diastolic or systolic diameter, respectively.

### Survival Rate

At the end of the first stage, on day 35, the survival rate between the control group and the chronic viral myocarditis group was compared. At the end of the second stage, on day 65, the survival rate of the treatment group and the untreated group was compared after 30 days of treatment with ivabradine.

### Myocardial Histopathology and Tissue Viral Titers

The heart weight (HW), body weight (BW) and the ratio of HW to BW (HW/BW) were calculated. The heart tissue was fixed in 4% paraformaldehyde, routinely processed, paraffin embedded, sectioned and stained. Masson’s trichrome stain, a collagen-specific stain for interstitial fibrosis, was used to quantitatively estimate the expression of collagen and the fibrosis area. Images were acquired by a light microscope connected to a digital video camera (Olympus, Tokyo, Japan). Five sections of each heart were scored by two observers blindly. The scores assigned to these specific sections were averaged, as described in our previous papers (Li-Sha et al., 2015, 2016). The extent of myocardial fibrosis was graded and scored as follows: 0 = no fibrosis; 1+ = fibrosis involving <25% of the myocardial interstitial; 2+ = fibrosis involving 25–50%; 3+ = fibrosis involving 50–75%; and 4+ = fibrosis involving 75% to 100%.

The viral titer of the myocardial tissue was estimated by the Reed–Muench method. Isolated cardiac tissue with aseptic conditions was washed with Hank’s liquid three times. Fragmentized myocardial tissue was digested by 0.25% trypsin for 5 min, and then virus maintenance fluid (DMEM: FBS = 98:2) was added to the digested myocyte suspension. The mixture was frozen at -20°C for 2 h and then thawed and shaken for 2 min to release the CVB3 in myocytes completely (repeated three times). Low temperature high speed centrifugation (4°C, 8000 r/min, 10 min) and filtration were performed. The Hep2 cell suspension was dropped into a cell culture plate, and the virus concentration gradient was diluted from 10^-1^ to 10^-7^. The Hep2 cells were cultured in an incubator at 37°C, 5% CO_2_ for 2 days.

### Western Blot

For the Western blot, proteins were separated on polyacrylamide gels and transferred to a PVDF membrane for detection with various antibodies, including primary monoclonal antibodies for tumor necrosis factor-α (TNF-α), interleukin-1β (IL-1β), interleukin-6 (IL-6), phospho-p38 MAPK (P-p38) and total-p38 MAPK (T-p38) (Cell Signaling Technology Corporation, USA) and a polyclonal antibody for collagen I, collagen III (Biorbyt Corporation, USA). The SDS-PAGE (8 and 12% polyacrylamide gels) method separated the protein, and electrophoresis (90 min, 80–120 V) and constant current (300 mA) transfer were used for the Western blots. The blots were blocked with 5% non-fat milk-TBST for 2 h at room temperature. The PVDF membrane was gently incubated overnight at 4°C with primary antibody (rabbit serum IgG) and incubated with the secondary antibody (HRP-marked goat anti-rabbit) for 1 h at room temperature and then exposed with ECL

### Statistical Analysis

Continuous variables are expressed as the mean ± standard deviation (SD). The survival rate was analyzed by the Kaplan–Meier method. Categories variables were performed by rank-sum test. Comparisons between groups were performed by one-way analysis of variance (ANOVA), followed by Fisher’s protected least significant difference test. A value was considered significant with *P* < 0.05.

## Results

### Mouse Model of Chronic Viral Myocarditis on Day 35

**FIGURE 2:**
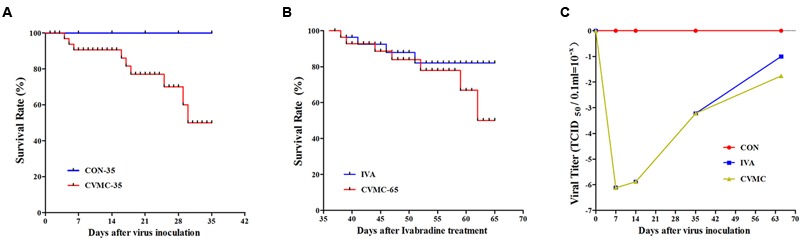
**The survival rate and cardiac tissue viral titer in CVB3-inoculated mice followed for 35 and 65 days. (A)** Survival rate on day 35. The CON-35 group (100%) versus the CVMC-35 group (47.69%). **(B)** Survival rate on day 65. IVA (80.00%) versus untreated CVMC-65 (48.57%). **(C)** Viral titer. IVA versus untreated CVMC-65, *P* = 0.286.

The characteristics of the chronic viral myocarditis mice are summarized in **Table 1**. No dead mice were found in the control group. The total survival rate of the chronic viral myocarditis mice on day 35 was 47.69% (62/130) (**Figure 2A**), and the LVEF in the chronic viral myocarditis mice (CVMC-35) was significantly lower than that in the normal control mice (CON-35) (*P* < 0.05). The expression of collagen and cytokines in the chronic myocarditis mice on day 35 was higher than that in the normal control groups (*P* < 0.05).

**Table 1:** Animal characteristics and cardiac function results.

Variable	CON-35	CVMC-35	CON-65	CVMC-65	IVA
**Mouse characteristics**		
Body weight, g	26.42 ± 1.963	19.9 ± 2.96	26.00 ± 3.65	22.09 ± 2.581	24.38 ± 3.70
Heart weight, mg	116.07 ± 7.948^☆^	95.32 ± 14.04^#^	117.7 ± 16.5	112.71 ± 10.50	104.13 ± 17.38
Heart weight/body weight, mg/g	4.40 ± 0.188^☆^	4.79 ± 0.193^#^	4.532 ± 0.197	5.13 ± 0.452^Δ^	4.28 ± 0.370^#^
**Cardiac function**		
Heart rate, bpm	492.2 ± 14.18	550.3 ± 22.52	505.4 ± 18.72	563.5 ± 14.15^∗^	412.7 ± 18.27^Δ^
Septum, mm	0.789 ± 0.018	0.662 ± 0.012	0.8 ± 0.013	0.613 ± 0.065	0.733 ± 0.0263
LVPW, mm	0.771 ± 0.023	0.668 ± 0.017	0.761 ± 0.046	0.654 ± 0.088	0.763 ± 0.0243^#^
LVEDd, mm	2.185 ± 0.107	2.97 ± 0.081	2.36 ± 0.079	3.637 ± 0.106^Δ^	2.389 ± 0.135^#^
LVESd, mm	1.31 ± 0.09	1.77 ± 0.103	1.46 ± 0.054	2.063 ± 0.185	1.328 ± 0.095^#^
LVEF, %	82.73 ± 1.66^☆^	71.00 ± 2.32	84.84 ± 1.402	66.07 ± 2.411^☆Δ^	82.24 ± 1.139^#^
FS, %	44.64 ± 0.455^☆^	36.52 ± 0.43	45.09 ± 0.344	34.55 ± 0.418^Δ^	40.88 ± 0.807^#^

*CON-35, normal control group 1; CVMC-35, chronic viral myocarditis group; CON-65, normal control group 2; CVMC-65, untreated chronic viral myocarditis group; IVA, ivabradine treatment group; LVPW, left ventricle posterior wall; LVESd, left ventricular end-systolic diameter; LVEDd, left ventricular end-diastolic diameter; LVEF, left ventricular ejection fraction; FS, fractional shortening; bpm, beats per minute. ^#^*P* < 0.05, versus CVMC-65; ^Δ^*P* < 0.05, versus CON-65; ^☆^*P* < 0.05, versus CVMC-35; ^∗^*P* < 0.05, versus IVA.*

The viral titer of myocardial tissue was estimated by the Reed–Muench method. The virus replication peak in the cardiac tissue was on days 4 to 7. On day 7 post-inoculation (p.i), the TCID_50_ = 10^-6.12±0.582^; On day 14 p.i, the TCID_50_ = 10^-5.88±0.655^; On day 35 p.i, the TCID_50_ = 10^-3.22±0.653^; In the control group, the TCID_50_ = 0. Ivabradine did not attenuate the viral titer in the cardiac tissue on day 65 compared with the untreated group (10^-1.00±0.091^ versus 10^-1.76±0.078^, *P* = 0.286) (**Figure 2C**).

### IVA Treatment in Chronic Viral Myocarditis Mice

#### Heart Enlargement and Fibrosis of Cardiac Tissue

**FIGURE 3:**
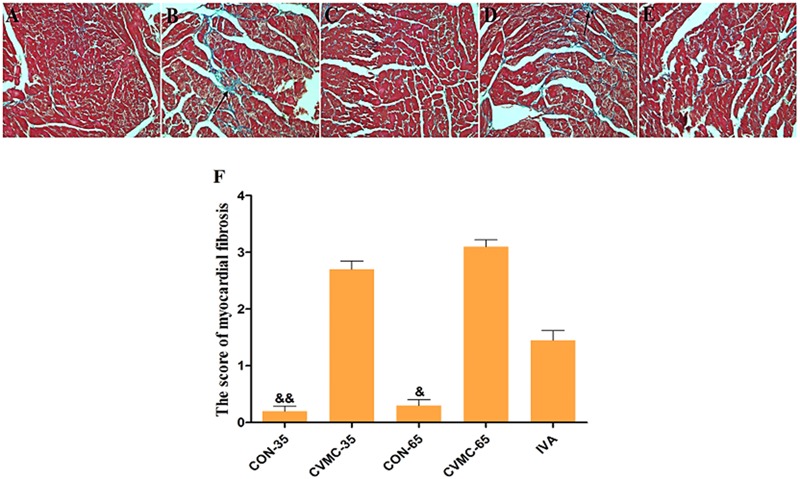
**Myocardial fibrosis in the heart (Masson’s Trichrome stain).** High power lens (40 × 10), fibrosis stained in blue (arrow), cardiac tissue stained in pink. **(A)** CON-35; **(B)** CVMC-35; **(C)** CON-65; **(D)** CVMC-65; **(E)** IVA; **(F)** Myocardial fibrosis scores. ^∗^*P* < 0.05 versus CVMC-65; ^&^*P* < 0.001 versus CVMC-65; ^&&^*P* < 0.001 versus CVMC-35.

On day 65, the survival rate of the treatment group (IVA) was 80.00% (16/20) with ivabradine (5 mg/kg per day) gavage for 30 days continuously (from day 35 to day 65), whereas the survival rate of the untreated group (CVMC-65) was 48.57% (17/35) (**Figure 2B**). Masson’s trichrome stain evaluated the fibrosis degree of the cardiac tissue (**Figures 3A–E**). The scores of myocardial fibrosis in the chronic viral myocarditis mice were significantly higher than in the CON-35 mice and the CON-65 mice. With the IVA treatment, the myocarditis mice showed significantly reduced myocardial fibrosis scores (*P* < 0.05). Myocardial fibrosis was increased in the CVMC-65 group compared with the CVMC-35 group. However, there was no significant difference between the two groups (*P* = 0.569) (**Figure 3F**).

#### HW/BW Ratio, Heart Rate, and Echocardiographic Findings

**FIGURE 4:**
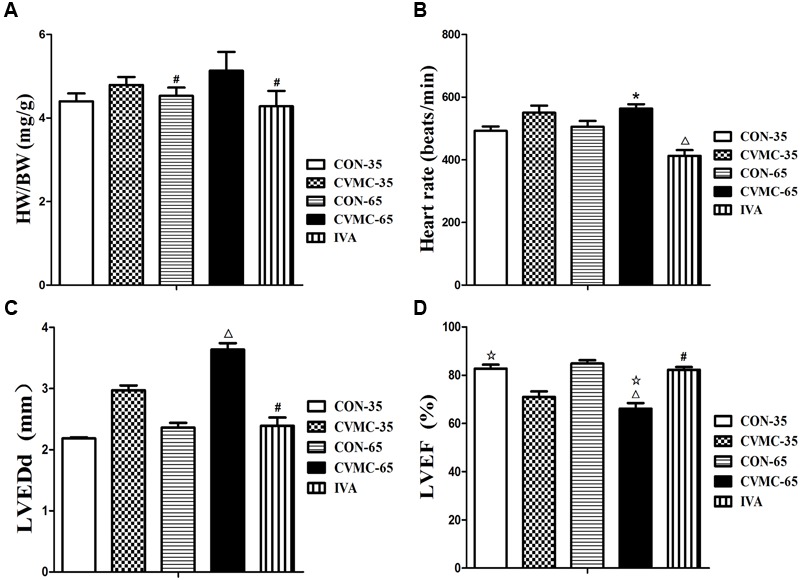
**Effects of ivabradine on HW/BW, heart rate, cardiac function and myocardial histopathology. (A)** HW/BW (The ratio of heart weight to body weight). ^#^*P* < 0.05, versus CVMC-65; **(B)** Heart rate. ^∗^*P* < 0.01, versus IVA; ^Δ^*P* < 0.001, versus CON-65; **(C)** LVEDd (left ventricular end-diastolic diameter). ^#^*P* < 0.05, versus CVMC-65; ^Δ^*P* < 0.05, versus CON-65; **(D)** LVEF (left ventricular ejection fraction). ^Δ^*P* < 0.05, versus CVMC-35; ^#^*P* < 0.05, versus CVMC-65; ^Δ^*P* < 0.05, versus CON-65.

The ratio of HW/BW was calculated. The echocardiographic findings of the chronic viral myocarditis mice are summarized in **Table 1**. As mentioned above, the CVMC-35 group and the untreated CVMC-65 group had a worse LVEF compared with the normal control group (*P <* 0.001), and the LVEF was significantly decreased (*P* < 0.05) (**Figure 4D**).

Ivabradine significantly reduced the ratio of HW/BW (*P* < 0.001) and HR (*P* < 0.01). The HW/BW ratio was significantly lower in the control groups than in the untreated CVMC 65 groups on day 65 (*P* < 0.05) (**Figure 4A**; **Table 1**). The HR of the IVA treatment mice was less than the control group (*P* < 0.001) (**Figure 4B**; **Table 1**). Ivabradine significantly enhanced the heart function of myocarditis mice comprehensively, improved the LVEF and FS (**Figure 4D**; **Table 1**). Ivabradine significantly decreased the size of the left ventricle (**Figure 4C**; **Table 1**). The LVEDD in the untreated CVMC-65 group was significantly larger than that in the control mice (*P* < 0.05) (**Figure 4C**; **Table 1**). The sizes of the chambers of the heart in untreated mice were larger than in the IVA treatment mice, and a thinner left ventricular wall was observed (**Table 1**). Despite the statistically significant difference in LVPW thickness between the IVA group and the CVMC-65 (*P* < 0.05), there is no statistically significant difference in septal wall thickness for these two groups (*P* = 0.075).

#### Ivabradine Reduces the Expression of Collagen and Cytokines

**FIGURE 5:**
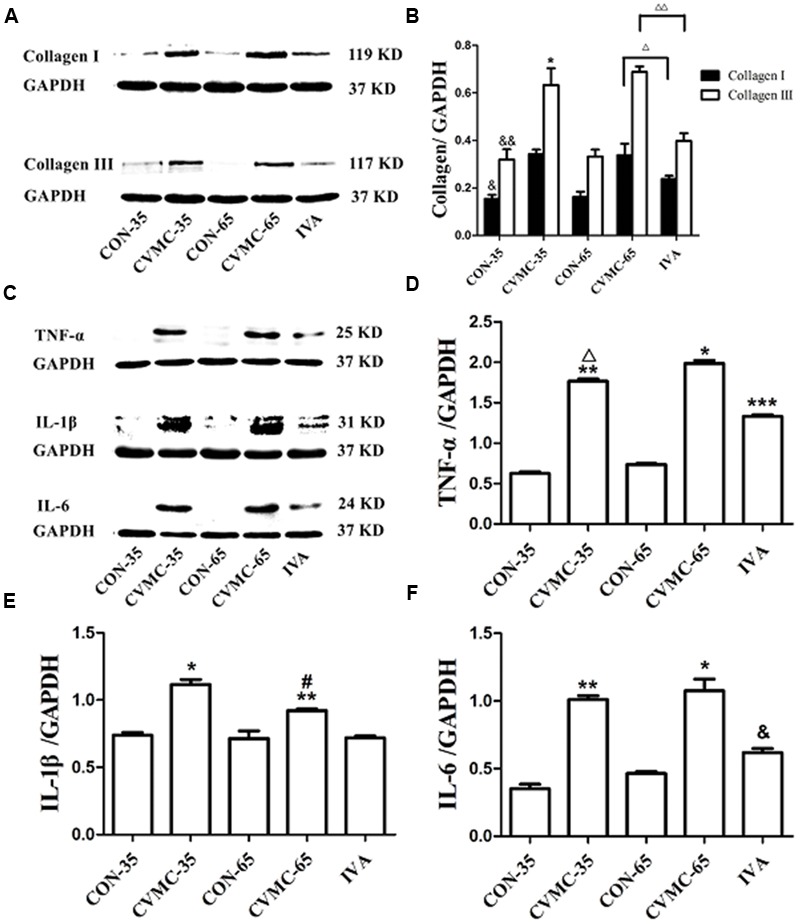
**Expression of collagens and cytokines in the myocardial tissues of mice on days 35 and 65. (A)** Western blot analysis for collagen I and collagen III. **(B)** Densitometric analysis of relative protein levels for collagen I and collagen III. ^Δ^*P* < 0.001, ^ΔΔ^*P* < 0.05, IVA versus CVMC-65; ^&^*P* < 0.05, ^&&^*P* < 0.05, versus CON-35; ^∗^*P* < 0.001, versus CVMC-65. **(C)** Western blot analysis for cytokines (TNF-α, IL-1β, and IL-6). **(D)** Densitometric analysis of relative protein levels for TNF-α. ^∗^*P* < 0.001, versus IVA; ^∗∗^*P* < 0.05, versus CON-35; ^∗∗∗^*P* < 0.05, versus CON-65; ^Δ^*P* < 0.05, versus CVMC-65. **(E)** Densitometric analysis of relative protein levels for IL-β. ^∗^*P* < 0.001, versus IVA; ^∗∗^*P* < 0.05, versus CON-35; ^#^*P* < 0.05, versus CVMC-65. **(F)** Densitometric analysis of relative protein levels for IL-6. ^∗^*P* < 0.001, versus IVA; ^&^*P* < 0.05, versus CON-65; ^∗∗^*P* < 0.05, versus CON-35.

Pathological sections showed the enlarged chambers in the chronic viral myocarditis mice, and ivabradine reversed ventricular remodeling and decreased the size of the heart. Collagen was the foremost index of fibrosis. Western blotting indicated that the production of collagen I and collagen III in the CVMC-35 mice was higher than that in the normal control group (*P* < 0.05, *P* < 0.05) (**Figures 5A,B**). The expression levels of collagen I and collagen III were significantly decreased in the ivabradine treatment group compared with the untreated group (both *P* < 0.05).

As shown in **Figures 5C–F**, Western blotting revealed that the expression of cytokines (TNF-α, IL-1β, and IL-6) in the CVMC-35 group was higher than that in the normal CON-35 group (all *P* < 0.05). Ivabradine significantly attenuated the expression of TNF-α, IL-1β and IL-6 in the treatment group compared with the untreated group (all *P* < 0.001). No difference was found among the CON-65 and IVA groups in the level of IL-1β (*P =* 0.181), although there were differences in TNF-α and IL-6 (*P* < 0.05).

#### The p38 MAPK Signaling Pathway

**FIGURE 6:**
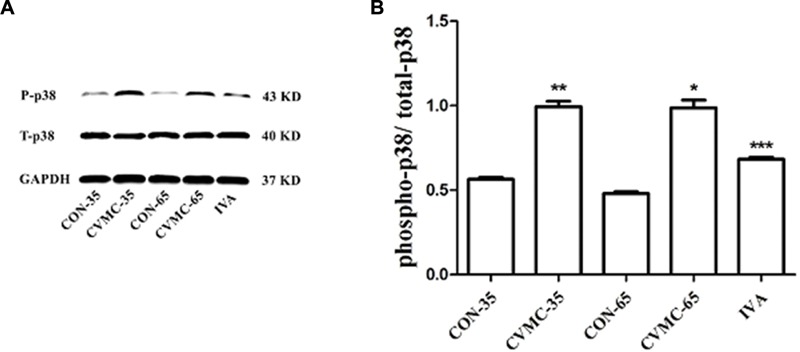
**Expression of p38 MAPK in the myocardial tissues of mice on days 35 and 65. (A)** Western blot analysis for phospho-p38 MAPK (P-p38) and total-p38 MAPK (T-p38). **(B)** Densitometric analysis of relative protein levels for P-p38/ T-p38. ^∗∗^*P* < 0.001, versus CON-35; ^∗∗∗^*P* < 0.001 versus untreated CVMC- 65; ^∗^*P* < 0.001 versus CON-65.

The ratio of phospho-p38 MAPK (P-p38) to total-p38 MAPK (T-p38) was calculated (**Figure 6**). There was no difference in the ratio of T-p38/GAPDH among the groups (*P* > 0.05). The P-p38 level in the CVMC-35 group was higher than that in the CON-35 group (*P* < 0.001), with the activation of the p38 MAPK signaling pathway.

The untreated CVMC-65 mice expressed a higher level of P-p38 than the CON-65 mice (*P* < 0.001), and ivabradine significantly attenuated the expression of P-p38 in the treatment group compared with the untreated group (*P* < 0.001).

#### The Progression of Chronic Viral Myocarditis

As the mouse model of chronic viral myocarditis progressed from day 35 to day 65, we found that there was increased fibrosis in both the CVMV-35 and CVMC-65 groups. However, there was no difference between the two groups (*P* = 0.569) (**Figure 3**). In the CVMC-65 mice and CVMC-35 mice, worse cardiac function occurred. The LVEF in the untreated group was markedly lower than that in the CVMC-35 group (*P* < 0.05) (**Table 1**). As time passed, no difference was observed in the level of collagen I between the untreated CVMC-65 and CVMC-35 groups (*P* = 0.184), although there was a difference in collagen III (*P* < 0.001) (**Figure 5**). No difference was found in the level of IL-6 between the untreated CVMC-65 and CVMC-35 groups (*P* = 0.204), although there were differences in TNF-α and IL-1β (*P* < 0.05) (**Figure 5**). There was no difference between the untreated CVMC-65 and CVMC-35 groups in the P-p38 expression levels (*P* = 0.659) (**Figure 6**).

## Discussion

The novel finding of the study is the marked amelioration of chronic viral myocarditis after treatment with ivabradine in a CVB3-induced murine chronic myocarditis model. To the best of our knowledge, this is the first study to investigate the effects of ivabradine in preventing progression from viral myocarditis to DCM. In this study, we demonstrated that ivabradine significantly inhibited the development of chronic viral myocarditis evolving to DCM. With the ivabradine treatment, the internal chamber dimension of the left ventricle was decreased, and both the LVEF and the thickness of the LVPW were increased markedly in CVB3-infected mice. Ivabradine significantly improved the survival rate and the impairment of the left ventricular function in the murine chronic viral myocarditis model. The findings indicated that ivabradine played a beneficial role in chronic viral myocarditis. Our previous studies demonstrated that ivabradine has a therapeutic benefit in an acute viral myocarditis mouse model (Yue-Chun et al., 2012; Li et al., 2013). The present study extends the understanding of ivabradine efficacy in murine viral myocarditis models (Yue-Chun et al., 2012; Li et al., 2013).

It is well-established that an increased HR is a risk factor for the development of cardiovascular diseases. HR-controlling treatments exert several beneficial effects. HR reduction decreases myocardial oxygen demand and improves endocardial blood supply. In addition, a prolonged left ventricular diastolic filling time improves left ventricular filling and inhibits sympathetic activity (Mulder et al., 2004; Li et al., 2013). In our previous study (Li et al., 2013) ivabradine significantly decreased the levels of plasma noradrenaline in mice with acute viral myocarditis on day 14. In rats with congestive HF, ivabradine significantly reduced noradrenaline levels on day 90 (Mulder et al., 2004). These studies indicated that the effect of ivabradine on plasma noradrenaline was not an acute effect, taking at least 2 weeks to develop. β-Blockade and the pure bradycardic agent ivabradine significantly reduced myocardial inflammation in murine acute viral myocarditis (Yue-Chun et al., 2012; Li et al., 2013; Li-Sha et al., 2015, 2016). Many experimental and clinical studies have demonstrated that ivabradine has a beneficial effect on left ventricular remodeling in the failing heart. In the present study, ivabradine significantly decreased HR and improved left ventricular remodeling. We propose that the main mechanism by which ivabradine exerts these effects is the reduction in HR because previous animal studies have shown that a reduction in HR by other means, eg, by ablation of the sinoatrial node in cynomolgus monkeys, beneficially affects the heart (Beere et al., 1984; Beere et al., 1992) and most effects of ivabradine can be reversed by atrial pacing (Heusch, 2008). However, this compound may have pleiotropic effects beyond HR reduction (Heusch, 2008). Therefore, long-term HR reduction induced by ivabradine is a useful target for preventing the progression toward myocardial dysfunction and preventing or even reversing remodeling in CVB3-infected chronic viral myocarditis mice.

Collagen is the main component of extracellular matrix (ECM). Collagen I and collagen III constitute 90% of the total collagen. The ECM is crucial for the structural integrity of the heart. Modulation of the balance between ECM-synthesis and ECM degradation is an important process of myocardial remodeling and left ventricular function. Moderate collagen production is a repair process under certain condition (Midwood et al., 2004; Chen and Raghunath, 2009). When synthesis exceeds degradation, an adverse accumulation of collagen appears to distort tissue structure. Its disproportionate accumulation, in the form of either a reactive or a reparative fibrosis, further increases stiffness and cardiac remodeling (Weber et al., 1994; Cohn et al., 2000). The common pathological basis of chronic viral myocarditis and DCM is the hyperplasia of collagen in the interstitium. The excessive accumulation of collagen is an important factor in the transition of acute myocarditis to DCM (Rutschow et al., 2010; Westermann et al., 2010). Rutschow et al. (2010) revealed that viral persistence induces chronic myocardial inflammation and significantly increases collagen production. Therefore, the reduction of the excessive collagen production is beneficial in preventing progression from viral myocarditis to DCM. In this study, collagen III was significantly increased in the untreated CVMC-65 mice compared with the untreated CVMC-35 mice (*P* < 0.001), although no difference was found in the expression of collagen I between the untreated CVMC-35 mice and CVMC-65 mice (*P* = 0.092). The secretion of collagen was increased gradually in the process of myocardial fibrosis, and the longer-term observation (e.g., up to 90 or 120 days after infection) might provide more meaningful data for collagen I. Collagen I and collagen III measured by Western blot were significantly decreased in the ivabradine treatment mice compared with the untreated CVMC-65 mice, which was consistent with the Masson’s Trichrome stain findings. With the treatment of ivabradine, the degree of fibrosis measured by Masson’s trichrome stain was significantly reduced (*P* < 0.05). A previous study (Rutschow et al., 2010) revealed that the high collagen content was associated with the development of left ventricular dysfunction and dilation in chronic viral myocarditis mice. In this study, ivabradine remarkably prevented the progression from viral myocarditis to DCM with myocardial collagen reduction. Echocardiography showed that the LVEF was significantly decreased in the CVMC-35 group compared with the CON-35 mice. On day 65, the LVEF was significantly decreased and the LVEDD was significantly increased in the CVMC-65 group compared with the CON-65 group. A lower LVEF was found in the untreated CVMC-65 group compared with the CVMC-35 group (*P* < 0.001). However, ivabradine significantly increased the LVEF (*P* < 0.001) and reduced the LVEDD (*P <* 0.05) in the treatment group compared with the untreated group. These results indicated that long-term HR reduction by ivabradine prevented the progression from viral myocarditis to DCM in mice with chronic viral myocarditis induced by coxsackievirus B3.

Based on the results of the Western blot, we found that cytokine levels were upregulated in CVMC-35 mice compared with the control mice (*P* < 0.05) and that the expression of the cytokines TNF-α, IL-1β, and IL-6 in the ivabradine treatment group was significantly less than in the untreated CVMC-65 mice (*P* < 0.001). A number of studies showed that cytokines play a vital role in tissue fibrosis (Fairweather et al., 2004; Liu et al., 2006). Gluck et al. (2001) suggested that the high level of cytokines in chronic viral myocarditis mice aggravates the damage of connective tissue. TNF-α increased ECM protein proliferation and induced the apoptosis and necrosis of myocardial cells, which are ultimately replaced or repaired by fibrous tissue. IL-6 and IL-1β directly induce myocardial fibrosis (Pulkki, 1997; Ono et al., 1998; Abreu et al., 2002; Eriksson et al., 2003). Based on the previous studies and the present study, we suggest that the effect of ivabradine on myocardial fibrosis is related to the reduction of the proinflammatory cytokines TNF-α, IL-1β, and IL-6.

The present study revealed that ivabradine could significantly inhibit activation of the p38 MAPK signaling pathway. Mitogen-activated protein kinase (MAPK) is an important signaling pathway that participates in multiple cellular processes, including inflammation, growth, differentiation, cell growth, and cell death (Clerk and Sugden, 2006). Myocardial hypertrophy, ventricle remodeling after myocardial infarction, myocardial apoptosis, inflammation, and atherosclerosis are also accompanied by activation of the MAPK pathway (Petrich and Wang, 2004; Streicher et al., 2010). p38 MAPK, one of the MAPK downstream components, plays an important role in inflammation, fibrosis, and apoptosis (Li et al., 2005). p38 MAPK could be induced by the stresses of inflammation, heat-shock protein, ultraviolet and specific antigen. Streicher et al. (2010) observed that with the activation of the p38 MAPK pathway, the transcription of genes for contractility, matrix remodeling, and inflammation was influenced. The activation of p38 MAPK is a necessary condition for cardiac remodeling for diabetic cardiomyopathy (Thandavarayan et al., 2009). Liao et al. (2001) suggested that normal mice had a better cardiac function than p38 MAPK transgenic mice. Petrich and Wang (2004) proved that the p38 pathway regulated the expression of the ECM and the deteriorated left ventricle remodeling. Based on the above mentioned studies, the p38 MAPK pathway might take part in the process of cardiac fibrosis of CVB3-induced chronic viral myocarditis, and down-regulating the expression of P-p38 might be helpful for reducing fibrosis, reversing ventricular remodeling and improving heart function. In this study, no difference was found in the expression of total-p38 in the five groups. However, the infected mice had a higher level of expression of P-p38 compared with the normal control mice. Ivabradine significantly attenuated the production of P-p38 compared with the untreated CVMC-65 group (*P* < 0.05). There was no difference between the CVMC-65 and CVMC-35 groups in P-p38 production (*P* = 0.659).

## Conclusion

The findings suggest a therapeutic effect of ivabradine in preventing the progression from viral myocarditis to DCM in mice with chronic viral myocarditis induced by coxsackievirus B3, is associated with inhibition of the p38 MAPK pathway, downregulated inflammatory responses and decreased collagen expression. Ivabradine appears a promising approach for the treatment of patients with viral myocarditis. Further experimental and clinical studies are welcome in the future.

## Author Contributions

YX and LY-C designed the experiment. LY-C, CG-Y, GL-S, XC, TX, LC, and DX-Y performed experiments. CG-Y and GL-S contributed to analysis the experimental data. LY-C and CG-Y wrote the paper.

## Conflict of Interest Statement

The authors declare that the research was conducted in the absence of any commercial or financial relationships that could be construed as a potential conflict of interest.
